# Efficient proximal gradient algorithm for inference of differential gene networks

**DOI:** 10.1186/s12859-019-2749-x

**Published:** 2019-05-02

**Authors:** Chen Wang, Feng Gao, Georgios B. Giannakis, Gennaro D’Urso, Xiaodong Cai

**Affiliations:** 1 Department of Electrical and Computer Engineering, University of Miami, 1251 Memorial Drive, Coral Gables, 33146 FL USA; 2 Department of Electrical and Computer Engineering, University of Minnesota, Minneapolis, 55455 MN USA; 3 Department of Molecular and Cellular Pharmacology, University of Miami, Miami, 33136 FL USA; 4 Sylvester Comprehensive Cancer Center, University of Miami, Miami, 33136 FL USA

**Keywords:** Gene network, Differential network, Proximal gradient method

## Abstract

**Background:**

Gene networks in living cells can change depending on various conditions such as caused by different environments, tissue types, disease states, and development stages. Identifying the differential changes in gene networks is very important to understand molecular basis of various biological process. While existing algorithms can be used to infer two gene networks separately from gene expression data under two different conditions, and then to identify network changes, such an approach does not exploit the similarity between two gene networks, and it is thus suboptimal. A desirable approach would be clearly to infer two gene networks jointly, which can yield improved estimates of network changes.

**Results:**

In this paper, we developed a proximal gradient algorithm for differential network (ProGAdNet) inference, that jointly infers two gene networks under different conditions and then identifies changes in the network structure. Computer simulations demonstrated that our ProGAdNet outperformed existing algorithms in terms of inference accuracy, and was much faster than a similar approach for joint inference of gene networks. Gene expression data of breast tumors and normal tissues in the TCGA database were analyzed with our ProGAdNet, and revealed that 268 genes were involved in the changed network edges. Gene set enrichment analysis identified a significant number of gene sets related to breast cancer or other types of cancer that are enriched in this set of 268 genes. Network analysis of the kidney cancer data in the TCGA database with ProGAdNet also identified a set of genes involved in network changes, and the majority of the top genes identified have been reported in the literature to be implicated in kidney cancer. These results corroborated that the gene sets identified by ProGAdNet were very informative about the cancer disease status. A software package implementing the ProGAdNet, computer simulations, and real data analysis is available as Additional file 1.

**Conclusion:**

With its superior performance over existing algorithms, ProGAdNet provides a valuable tool for finding changes in gene networks, which may aid the discovery of gene-gene interactions changed under different conditions.

**Electronic supplementary material:**

The online version of this article (10.1186/s12859-019-2749-x) contains supplementary material, which is available to authorized users.

## Background

Genes in living cells interact and form a complex network to regulate molecular functions and biological processes. Gene networks can undergo topological changes depending on the molecular context in which they operate [1, 2]. For example, it was observed that transcription factors (TFs) can bind to and thus regulate different target genes under varying environmental conditions [3, 4]. Changes of genetic interactions when cells are challenged by DNA damage as observed in [5] may also reflect the structural changes of the underlying gene network. This kind of rewiring of gene networks has been observed not only in yeast [3–6], but also in mammalian cells [7, 8]. More generally, differential changes of gene networks can occur depending on environment, tissue type, disease state, development and speciation [1]. Therefore, identification of such differential changes in gene networks is of paramount importance when it comes to understanding the molecular basis of various biological processes.

Although a number of computational methods have been developed to infer the structure of gene regulatory networks from gene expression and related data [9–12], they are mainly concerned with the static structure of gene networks under a single condition. These methods rely on similarity measures such as the correlation or mutual information [13, 14], Gaussian graphical models (GGMs) [15, 16], Bayesian networks [17, 18], or linear regression models [19–22]. Refer to [12] for description of more network inference methods and their performance. Existing methods for the analysis of *differential* gene interactions under different conditions typically attempt to identify differential co-expression of genes based on correlations between their expression levels [23]. While it is possible to use an existing method to infer a gene network under different conditions separately, and then compare the inferred networks to determine their changes, such an approach does not jointly leverage the data under different conditions in the inference; thus, it may markedly sacrifice the accuracy in the inference of network changes.

In this paper, we develop a very efficient proximal gradient algorithm for differential network (ProGAdNet) inference, that jointly infers gene networks under two different conditions and then identifies changes in these two networks. To overcome the challenge of the small sample size and a large number of unknowns, which is common to inference of gene networks, our method exploits two important attributes of gene networks: i) sparsity in the underlying connectivity, meaning that the number of gene-gene interactions in a gene network is much smaller than the number of all possible interactions [19, 24–26]; and, ii) similarity in the gene networks of the same organism under different conditions [4, 7], meaning that the number of interactions changed in response to different conditions is much smaller than the total number of interactions present in the network. A similar network inference setup was considered in [27] for inferring multiple gene networks, but no new algorithm was developed there; instead [27] adopted the lqa algorithm of [28] that was designed for generalized linear models. Our computer simulations demonstrated superior performance of our ProGAdNet algorithm relative to existing methods including the lqa algorithm. Analysis of a set of RNA-Seq data from normal tissues and breast tumors with ProGAdNet identified genes involved in changes of the gene network.

The differential gene-gene interactions identified by our ProGAdNet algorithm yield a list of genes that may not be differentially expressed under two different conditions. Comparing with the set of differentially expressed genes under two conditions, this set of genes may provide additional insight into the molecular mechanism behind the phenotypical difference of the tissue under different conditions. Alternatively, the two gene networks inferred by our ProGAdNet algorithm can be used for further differential network analysis (DiNA). DiNA has received much attention recently; the performance of ten DiNA algorithms was assessed in [29] using gene networks and gene/microRNA networks. Given two networks with the same set of nodes, a DiNA algorithm computes a score for each node based on the difference of global and/or local topologies of the two networks, and then ranks nodes based on these scores. Apparently, DiNA relies on the two networks that typically need to be constructed from certain data. Our ProGAdNet algorithm provides an efficient and effective tool for constructing two gene networks of the same set of genes from gene expression data under two different conditions, which can be used by a DiNA algorithm for further analysis.

## Methods

### Gene network model

Suppose that expression levels of *p* genes have been measured with microarray or RNA-seq, and let *X*_*i*_ be the expression level of the *i*th gene, where *i*=1,…,*p*. To identify the possible regulatory effect of other genes on the *i*th gene, we employ the following linear regression model as also used in [19–22]

1 $$ X_{i}=\mu_{i}+\sum\limits_{j=1, j\ne i}^{p} X_{j} b_{ji}+E_{i},  $$

where *μ*_*i*_ is a constant and *E*_*i*_ is the error term, and unknown regression coefficients (*b*_*ji*_)’s reflect the correlation between *X*_*i*_ and *X*_*j*_ after adjusting the effects of other variables, *X*_*k*_’s, *k*∉{*i,j*}. This adjusted correlation may be the result of possible interaction between genes *i* and *j*. The nonzero (*b*_*ji*_)’s define the edges in the gene network. Suppose that *n* samples of gene expression levels of the same organism (or the same type of tissue of an organism) under two different conditions are available, and let *n*×1 vectors **x**_*i*_ and $\tilde {\mathbf {x}}_{i}$ contain these *n* samples of the *i*th gene under two conditions, respectively. Define *n*×*p* matrices **X**:=[**x**_1_,**x**_2_,…,**x**_*p*_] and $\tilde {\mathbf {X}}:=[\tilde {\mathbf {x}}_{1}, \tilde {\mathbf {x}}_{2}, \ldots, \tilde {\mathbf {x}}_{p}], p\times 1$ vectors ***μ***=[*μ*_1_,…,*μ*_*p*_]^*T*^ and $\tilde {\boldsymbol {\mu }}=[\tilde {\mu }_{1},\ldots, \tilde {\mu }_{p}]^{T}$, and *p*×*p* matrices **B** and $\tilde {\mathbf {B}}$ whose element on the *i*th column and the *j*th row are *b*_*ji*_ and $\tilde {b}_{ji}$, respectively. Letting $b_{ii}=\tilde {b}_{ii}=0$, model (1) yields the following

2 $$ \begin{aligned} \mathbf{X}&=\mathbf{1}\boldsymbol{\mu}^{T}+\mathbf{X}\mathbf{B} +\mathbf{E}\\ \tilde{\mathbf{X}}&=\mathbf{1}\tilde{\boldsymbol{\mu}}^{T}+\tilde{\mathbf{X}}\tilde{\mathbf{B}}+\tilde{\mathbf{E}}, \end{aligned}  $$

where **1** is a vector with all elements equal to 1, and *n*×*p* matrices **E** and $\tilde {\mathbf {E}}$ contain error terms. Matrices **B** and $\tilde {\mathbf {B}}$ characterize the structure of the gene networks under two conditions.

Our main goal is to identify the changes in the gene network under two conditions, namely, those edges from gene *j* to gene *i* such that $b_{ji}-\tilde {b}_{ji}\ne 0, j\ne i$. One straightforward way to do this is to estimate **B** and $\tilde {\mathbf {B}}$ separately from two linear models in (2), and then find gene pairs (*i,j*) for which $b_{ji}-\tilde {b}_{ji}\ne 0$. However, this approach may not be optimal, since it does not exploit the fact that the network structure does not change significantly under two conditions, that is, most entries of **B** and $\tilde {\mathbf {B}}$ are identical. A better approach is apparently to infer gene networks under two conditions jointly, which can exploit the similarity between two network structures and thereby improve the inference accuracy.

If we denote the *i*th column of **B** and $\tilde {\mathbf {B}}$ as **b**_*i*_ and $\tilde {\mathbf {b}}_{i}$, we can also write model (2) for each gene separately as follows: **x**_*i*_=*μ*_*i*_**1**+**Xb**_*i*_+**e**_*i*_ and $\tilde {\mathbf {x}}_{i}=\tilde {\mu }_{i}\mathbf {1}+\tilde {\mathbf {X}}\tilde {\mathbf {b}}_{i}+\tilde {\mathbf {e}}_{i}, i=1,\ldots, p$, where **e**_*i*_ and $\tilde {\mathbf {e}}_{i}$ are the *i*th column of **E** and $\tilde {\mathbf {E}}$, respectively. We can estimate *μ*_*i*_ and $\tilde {\mu }_{i}$ using the least square estimation method and substitute the estimates into the linear regression model, which is equivalent to centering each column of **X** and $\tilde {\mathbf {X}}$, i.e., subtracting the mean of each column from each element of the column. From now on, we will drop *μ*_*i*_ and $\tilde {\mu }_{i}$ from the model and assume that columns of **X** and $\tilde {\mathbf {X}}$ have been centered. To remove the constraints *b*_*ii*_=0,*i*=1,…,*p*, we define matrices **X**_−*i*_:=[**x**_1_,…,**x**_*i*−1_,**x**_*i*+1_,…,**x**_*p*_] and $\tilde {\mathbf {X}}_{-i}:=[\tilde {\mathbf {x}}_{1}, \ldots, \tilde {\mathbf {x}}_{i-1}, \tilde {\mathbf {x}}_{i+1}, \ldots, \tilde {\mathbf {x}}_{p}]$, vectors ***β***_*i*_:=[*b*_1*i*_,…,*b*_(*i*−1)*i*_,*b*_(*i*+1)*i*_,…,*b*_*pi*_]^*T*^ and $\tilde {\boldsymbol {\beta }}_{i}:=[\tilde {b}_{1i}, \ldots, \tilde {b}_{(i-1)i}, \tilde {b}_{(i+1)i}, \ldots, \tilde {b}_{pi}]^{T}$. The regression model for the gene network under two conditions can be written as

3 $$ \begin{aligned} \mathbf{x}_{i}&=\mathbf{X}_{-i}\boldsymbol{\beta}_{i}+\mathbf{e}_{i}\\ \tilde{\mathbf{x}}_{i}&=\tilde{\mathbf{X}}_{-i}\tilde{\boldsymbol{\beta}}_{i}+\tilde{\mathbf{e}}_{i}, \; i=1,\ldots,p. \end{aligned}  $$

Based on (3), we will develop a proximal gradient algorithm to infer ***β***_*i*_ and $\tilde {\boldsymbol {\beta }}_{i}$ jointly, and identify changes in the network structure.

### Network inference

#### Optimization formulation

As argued in [19, 30, 31], gene regulatory networks or more general biochemical networks are sparse, meaning that a gene directly regulates or is regulated by a small number of genes relative to the total number of genes in the network. Taking into account sparsity, only a relatively small number of entries of **B** and $\tilde {\mathbf {B}}$, or equivalently entries of ***β***_*i*_ and $\tilde {\boldsymbol {\beta }}_{i}, i=1,\ldots, p$, are nonzero. These nonzero entries determine the network structure and the regulatory effect of one gene on other genes. As mentioned earlier, the gene network of an organism is expected to have similar structure under two different conditions. For example, the gene network of a tissue in a disease (such as cancer) state may have changed, comparing to that of the same tissue under the normal condition, but such change in the network structure is expected to be small relative to the overall network structure. Therefore, it is reasonable to expect that the number of edges that change under two conditions is small comparing with the total number of edges of the network.

Taking into account sparsity in **B** and $\tilde {\mathbf {B}}$ and also the similarity between **B** and $\tilde {\mathbf {B}}$, we formulate the following optimization problem to jointly infer gene networks under two conditions:

4 $$ \begin{aligned} \left(\hat{\boldsymbol{\beta}_{i}}, \hat{\tilde{\boldsymbol{\beta}}}_{i}\right)=&\text{arg min}_{\boldsymbol{\beta}_{i},\tilde{\boldsymbol{\beta}}_{i}} \left\{\parallel \mathbf{x}_{i}-\mathbf{X}_{-i}\boldsymbol{\beta}_{i}\parallel^{2}\right. \\ &+ \parallel \tilde{\mathbf{x}}_{i}-\tilde{\mathbf{X}}_{-i}\tilde{\boldsymbol{\beta}}_{i}\parallel^{2}+\lambda_{1}(\parallel\boldsymbol{\beta}_{i}\parallel_{1}+\parallel\tilde{\boldsymbol{\beta}}_{i}\parallel_{1})\\ &+\left.\lambda_{2}\parallel\boldsymbol{\beta}_{i}-\tilde{\boldsymbol{\beta}}_{i}\parallel_{1}\right\}, \end{aligned}  $$

where ∥·∥ stands for Euclidean norm, ∥·∥_1_ stands for *l*_1_ norm, and *λ*_1_ and *λ*_2_ are two positive constants. The objective function in (4) consists of the squared error of the linear regression model (1) and two regularization terms $\lambda _{1}(\parallel \boldsymbol {\beta }_{i}\parallel _{1}+\parallel \tilde {\boldsymbol {\beta }}_{i}\parallel _{1})$ and $\lambda _{2}\parallel \boldsymbol {\beta }_{i}-\tilde {\boldsymbol {\beta }}_{i}\parallel _{1}$. Note that unlike the GGM, the regularized least squared error approach here does not rely on the Gaussian assumption. The two regularization terms induce sparsity in the inferred networks and network changes, respectively. This optimization problem is apparently convex, and therefore it has a unique and globally optimal solution. Note that the term $\lambda _{2}\parallel \boldsymbol {\beta }_{i}-\tilde {\boldsymbol {\beta }}_{i}\parallel _{1}$ is reminiscent of the fused Lasso [32]. However, all regression coefficients in the fused Lasso are essentially coupled, whereas here the term $\lambda _{2}\parallel \boldsymbol {\beta }_{i}-\tilde {\boldsymbol {\beta }}_{i}\parallel _{1}$ only couples each pair of regression coefficients, *β*_*ij*_ and $\tilde {\beta }_{ij}$. As will be described next, this enables us to develop an algorithm to solve optimization problem (4) that is different from and more efficient than the algorithm for solving the general fused Lasso problem. Note that an optimization problem similar to (4) was formulated in [27] for inferring multiple gene networks, but no new algorithm was developed, instead the problem was solved with the lqa algorithm [28] that was developed for general penalized maximum likelihood inference of generalized linear models including the fused Lasso. Our computer simulations showed that our algorithm not only is much faster than the lqa algorithm, but also yields much more accurate results.

#### Proximal Gradient Solver

Define $\boldsymbol {\alpha }_{i}:=\left [\boldsymbol {\beta }_{i}^{T} ~\tilde {\boldsymbol {\beta }}_{i}^{T} \right ]^{T}$, and let us separate the objective function in (4) into the differentiable part *g*_1_(***α***_*i*_) and the non-differentiable part *g*_2_(***α***_*i*_) given by

5 $$ \begin{aligned} g_{1}(\boldsymbol{\alpha}_{i}) &= \parallel \mathbf{x}_{i}-\mathbf{X}_{-i}\boldsymbol{\beta}_{i}\parallel^{2} +\parallel \tilde{\mathbf{x}}_{i}-\tilde{\mathbf{X}}_{-i}\tilde{\boldsymbol{\beta}}_{i}\parallel^{2},\\ g_{2}(\boldsymbol{\alpha}_{i}) &= \lambda_{1}(\parallel\boldsymbol{\beta}_{i}\parallel_{1}+\parallel\tilde{\boldsymbol{\beta}}_{i}\parallel_{1})+\lambda_{2}\parallel\boldsymbol{\beta}_{i}-\tilde{\boldsymbol{\beta}}_{i}\parallel_{1}. \end{aligned}  $$

Applying the proximal gradient method [33] to solve the optimization problem (4), we obtain an expression for ***α***_*i*_ in the *r*th step of the iterative procedure as follows:

6 $$ \boldsymbol{\alpha}_{i}^{(r+1)}=\text{prox}_{\lambda^{(r)}g_{2}}[\boldsymbol{\alpha}_{i}-\lambda^{(r)}\nabla g_{1}(\boldsymbol{\alpha}_{i})],  $$

where prox stands for the proximal operator defined as $\text {prox}_{\lambda f}(\mathbf {t}):=\text {arg min}_{\mathbf {x}} f(\mathbf {x})+\frac {1}{2\lambda }||\mathbf {x}-\mathbf {t}||^{2}$ for function *f*(**x**) and a constant vector **t**, and ∇*g*_1_(***α***_*i*_) is the gradient of *g*_1_(***α***_*i*_). Generally, the value of step size *λ*^(*r*)^ can be found using a line search step, which can be determined from the Lipschitz constant [33]. For our problem, we will provide a closed-form expression for *λ*^(*r*)^ later. Since *g*_1_(***α***_*i*_) is simply in a quadratic form, its gradient can be obtained readily as $\nabla g_{1}(\boldsymbol {\alpha }_{i})= \left [\nabla g_{1}(\boldsymbol {\beta }_{i})^{T}, \nabla g_{1}(\tilde {\boldsymbol {\beta }}_{i})^{T}\right ]^{T}$, where $\nabla g_{1}(\boldsymbol {\beta }_{i})=2\left (\mathbf {X}_{-i}^{T}\mathbf {X}_{-i}\boldsymbol {\beta }_{i}-\mathbf {X}_{-i}^{T}\mathbf {x}_{i}\right)$ and $\nabla g_{1}(\tilde {\boldsymbol {\beta }}_{i})=2\left (\tilde {\mathbf {X}}_{-i}^{T}\tilde {\mathbf {X}}_{-i}\tilde {\boldsymbol {\beta }}_{i}-\tilde {\mathbf {X}}_{-i}^{T}\tilde {\mathbf {x}}_{i}\right)$.

Upon defining **t**=***β***_*i*_−*λ*^(*r*)^∇*g*_1_(***β***_*i*_) and $\tilde {\mathbf {t}}=\tilde {\boldsymbol {\beta }}_{i}-\lambda ^{(r)}\nabla g_{1}(\tilde {\boldsymbol {\beta }}_{i})$, the proximal operator in (6) can be written as

7 $$ \begin{aligned} \text{prox}_{\lambda^{(r)}g_{2}}(\mathbf{t})=\text{arg min}_{\boldsymbol{\beta}_{i}, \tilde{\boldsymbol{\beta}}_{i}} \left\{{\vphantom{\frac{1}{2\lambda^{(r)}}}}\lambda_{1}(\parallel\boldsymbol{\beta}_{i}\parallel_{1}+\parallel\tilde{\boldsymbol{\beta}}_{i}\parallel_{1}) +\lambda_{2}\parallel\boldsymbol{\beta}_{i}-\tilde{\boldsymbol{\beta}}_{i}\parallel_{1}\right.\\ \left.+\frac{1}{2\lambda^{(r)}}\left(\parallel \boldsymbol{\beta}_{i}-\mathbf{t}\parallel^{2} +\parallel \tilde{\boldsymbol{\beta}}_{i}-\tilde{\mathbf{t}}\parallel^{2}\right)\right\}. \end{aligned}  $$

It is seen that the optimization problem in proximal operator (7) can be decomposed into *p*−1 separate problems as follows

8 $$ \begin{aligned} \text{arg min}_{\beta_{ij}, \tilde{\beta}_{ij}} \left\{{\vphantom{\frac{1}{2\lambda^{(r)}}}}\lambda_{1}(|\beta_{ij}|+|\tilde{\beta}_{ij}|) +\lambda_{2}|\beta_{ij}-\tilde{\beta}_{ij}|\right.\\ \left. +\frac{1}{2\lambda^{(r)}}\left((\beta_{ij}-t_{j})^{2}+(\tilde{\beta}_{ij}-\tilde{t}_{j})^{2}\right)\right\}\\ j=1,\ldots, p-1, \end{aligned}  $$

where *β*_*ij*_ and $\tilde {\beta }_{ij}$ are the *j*th element of ***β***_*i*_ and $\tilde {\boldsymbol {\beta }}_{i}$, respectively, and *t*_*j*_ and $\tilde {t}_{j}$ are the *j*th element of **t** and $\tilde {\mathbf {t}}$, respectively. The optimization problem (8) is in the form of the fused Lasso signal approximator (FLSA) [34]. The general FLSA problem has many variables, and numerical optimization algorithms were developed to solve the FLSA problem [34, 35]. However, our problem has only two variables, which enables us to find the solution of (8) in closed form. This is then used in each step of our proximal gradient algorithm for network inference.

Let us define a soft-thresholding operator *S*(*x,a*) as follows

9 $$  S(x,a)= \left\{ \begin{aligned} x-a, & ~\text{if} ~x>a\\ x+a, &~\text{if} ~x<-a\\ 0, &~\text{otherwise}, \end{aligned} \right.  $$

where *a* is a positive constant. Then as shown in [34], if the solution of (8) at *λ*_1_=0 is $\hat \beta _{ij}^{(0)}$ and $\hat {\tilde \beta }_{ij}^{(0)}$, the solution of (8) at *λ*_1_>0 is given by

10 $$ \begin{aligned} \hat\beta_{ij}&=S\left(\hat{\beta}_{ij}^{(0)}-\tilde\lambda_{1}\right)\\ \hat{\tilde\beta}_{ij}&=S\left(\hat{\tilde\beta}_{ij}^{(0)},\tilde\lambda_{1}\right), \end{aligned}  $$

where $\tilde \lambda _{1}=\lambda _{1} \lambda ^{(r)}$. Therefore, if we can solve the problem (8) at *λ*_1_=0, we can find the solution of (8) at any *λ*_1_>0 from (10). It turns out that the solution of (8) at *λ*_1_=0 can be found as

11 $$  \left({\hat\beta}^{(0)}_{ij}, \hat{\tilde\beta}^{(0)}_{ij}\right)= \left\{ \begin{aligned} \left(\frac{t_{j}+\tilde{t}_{j}}{2},\frac{t_{j}+\tilde{t}_{j}}{2}\right), &~\text{if}~ |t_{j}-\tilde{t}_{j}| \leq 2\tilde\lambda_{2}\\ (t_{j}-\tilde\lambda_{2}, \tilde{t}_{j}+\tilde\lambda_{2}), &~\text{if}~ t_{j}-\tilde{t}_{j} > 2\tilde\lambda_{2}\\ (t_{j}+\tilde\lambda_{2}, \tilde{t}_{j}-\tilde\lambda_{2}), &~\text{otherwise}, \end{aligned} \right.  $$

where $\tilde \lambda _{2}=\lambda _{2} \lambda ^{(r)}$. Therefore, our proximal gradient method can solve the network inference problem (6) efficiently through an iterative process, where each step of the iteration solves the optimization problem (6) in closed form specified by (10) and (11). To obtain a complete proximal gradient algorithm, we need to find the step size *λ*^(*r*)^ as will be described next.

#### Stepsize

As mentioned in [33], if the step size *λ*^(*r*)^∈[0,1/*L*], where *L* is the Lipschitz constant of ∇*g*_1_(***α***_*i*_), then the proximal gradient algorithm converges to yield the optimal solution. We next derive an expression for *L*. Specifically, we need to find *L* such that $\parallel \nabla g_{1}\left (\boldsymbol {\alpha }_{i}^{(1)}\right)-\nabla g_{1}\left (\boldsymbol {\alpha }_{i}^{(2)}\right)\parallel _{2}\le L\parallel \left (\boldsymbol {\alpha }_{i}^{(1)}-\boldsymbol {\alpha }_{i}^{(2)}\right)\parallel _{2}$ for any $\boldsymbol {\alpha }_{i}^{(1)}\ne \boldsymbol {\alpha }_{i}^{(2)}$, which is equivalent to

12 $$  2\left\|\begin{array}{cc} \mathbf{X}_{-i}^{T}\mathbf{X}_{-i}\left(\boldsymbol{\beta}_{i}^{(1)}-\boldsymbol{\beta}_{i}^{(2)}\right)\\ \tilde{\mathbf{X}}_{-i}^{T}\tilde{\mathbf{X}}_{-i}\left(\tilde{\boldsymbol{\beta}}_{i}^{(1)}-\tilde{\boldsymbol{\beta}}_{i}^{(2)}\right) \end{array}\right\| \leq L \left\|\begin{array}{cc} \boldsymbol{\beta}_{i}^{(1)}-\boldsymbol{\beta}_{i}^{(2)} \\ \tilde{\boldsymbol{\beta}}_{i}^{(1)}-\tilde{\boldsymbol{\beta}}_{i}^{(2)} \end{array}\right\|  $$

for any $\left (\boldsymbol {\beta }_{i}^{(1)}, \tilde {\boldsymbol {\beta }}_{i}^{(1)}\right)\ne \left (\boldsymbol {\beta }_{i}^{(2)}, \tilde {\boldsymbol {\beta }}_{i}^{(2)}\right)$. Let *γ* and $\tilde \gamma $ be the maximum eigenvalues of $\mathbf {X}_{-i}^{T}\mathbf {X}_{-i}$ and $\tilde {\mathbf {X}}_{-i}^{T}\tilde {\mathbf {X}}_{-i}$, respectively. It is not difficult to see that (12) will be satisfied if $L=2(\gamma +\tilde \gamma)$. Note that $\mathbf {X}_{-i}^{T}\mathbf {X}_{-i}$ and $\mathbf {X}_{-i}\mathbf {X}_{-i}^{T}$ have the same set of eigenvalues. And thus, *γ* can be found using a numerical algorithm with a computational complexity of *O*((min(*n,p*))^2^). After obtaining *L*, the step size of our proximal gradient algorithm can be chosen to be *λ*^(*r*)^=1/*L*. Note that *λ*^(*r*)^ does not change across iterations, and it only needs to be computed once. Since the sum of the eigenvalues of a matrix is equal to the trace of matrix, another possible value for *L* is $2\left (\text {trace}\left (\mathbf {X}_{-i}^{T}\mathbf {X}_{-i}\right)+\text {trace}\left (\tilde {\mathbf {X}}_{-i}^{T}\tilde {\mathbf {X}}_{-i}\right)\right)$, which can save the cost of computing *γ* and $\tilde \gamma $. However, this value of *L* is apparently greater than $2(\gamma +\tilde \gamma)$, which reduces the step size *λ*^(*r*)^, and may affect the convergence speed of the algorithm.

#### Algorithm

The proximal gradient solver of (4) for inference of differential gene networks is abbreviated as ProGAdNet, and is summarized in the following table.


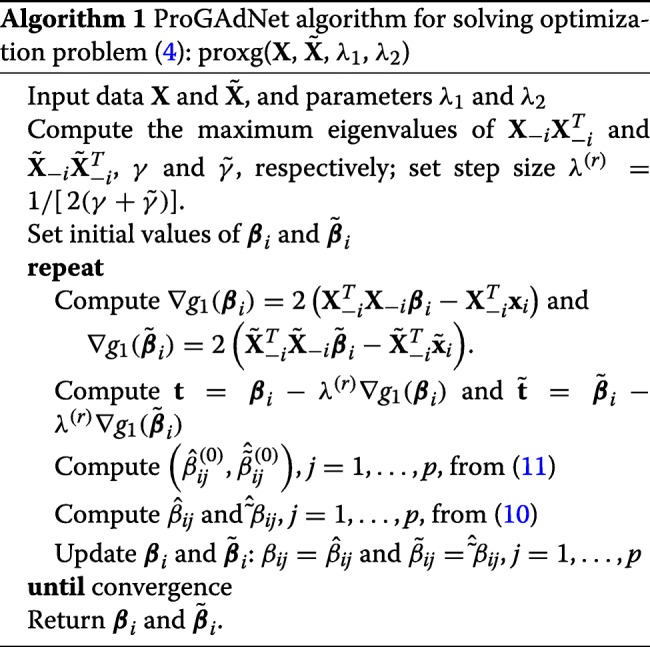


#### Maximum values of *λ*_1_ and *λ*_2_

The ProGAdNet solver of (4) is outlined in Algorithm 1 with a specific pair of values of *λ*_1_ and *λ*_2_. However, we typically need to solve the optimization problem (4) over a set of values of *λ*_1_ and *λ*_2_, and then either use cross validation to determine the optimal values of *λ*_1_ and *λ*_2_, or use the stability selection technique to determine nonzero elements of ***β***_*i*_ and $\tilde {\boldsymbol {\beta }}_{i}$, as will be described later. Therefore, we also need to know the maximum values of *λ*_1_ and *λ*_2_. In the following, we will derive expressions for the maximum values of *λ*_1_ and *λ*_2_.

When we determine the maximum values of *λ*_1_ (denoted as *λ*_1 max_), *λ*_2_ can be omitted in our optimization problem, since when *λ*_1_=*λ*_1 max_, we have *β*_*ij*_=0 and $\tilde {\beta }_{ij} = 0, \forall i$ and *j*. Thus, we can use the same method for determining the maximum value of *λ* in the Lasso problem [36] to find *λ*_1 max_, which leads to

13 $$ \lambda_{1\max} = \max\left\{\max_{j \neq i} 2{|\mathbf{x}_{j}^{T}\mathbf{x}_{i}}|, \max_{j \neq i} 2{|\tilde{\mathbf{x}}_{j}^{T}\tilde{\mathbf{x}}_{i}}|\right\}.  $$

The maximum value of *λ*_2_,*λ*_2 max_ depends on *λ*_1_. It is difficult to find *λ*_2 max_ exactly. Instead, we will find an upper-bound for *λ*_2 max_. Let us denote the objective function in (4) as $J(\boldsymbol {\beta }_{i}, \tilde {\boldsymbol {\beta }}_{i})$, and let the *j*th column of **X**_−*i*_ ($\tilde {\mathbf {X}}_{-i}$) be **z**_*i*_ ($\tilde {\mathbf {z}}_{i}$). If the optimal solution of (4) is $\boldsymbol {\beta }_{i}=\tilde {\boldsymbol {\beta }}_{i}=\boldsymbol {\beta }^{*}$, then the subgradient of $J(\boldsymbol {\beta }_{i}, \tilde {\boldsymbol {\beta }}_{i})$ at the optimal solution should contain the zero vector, which yields

14 $$ \begin{aligned} &2\mathbf{z}_{j}^{T}\left(\mathbf{x}_{i}-\mathbf{X}_{-i}\boldsymbol{\beta}^{*}\right)+\lambda_{1}s_{1j}+\lambda_{2}s_{2j}\,=\,0, \; j=1,\ldots, p-1\\ &2\tilde{\mathbf{z}}_{j}^{T}\left(\tilde{\mathbf{x}}_{i}-\tilde{\mathbf{X}}_{-i}\boldsymbol{\beta}^{*}\right)\!+\lambda_{1}\tilde{s}_{1j}+\lambda_{2}\tilde{s}_{2j}\,=\,0, \; j\,=\,1,\ldots, p-1, \end{aligned}  $$

where *s*_1*j*_=1 if *β*_*ij*_>0,=−1 if *β*_*ij*_<0, or ∈ [−1,1] if *β*_*ij*_=0, and *s*_2*j*_∈ [−1,1], and similarly, $\tilde {s}_{1j}=1$ if $\tilde {\beta }_{ij}>0, =-1$ if $\tilde {\beta }_{ij}<0$, or ∈[−1,1] if $\tilde {\beta }_{ij}=0$, and $\tilde {s}_{2j}\in [-1, 1]$. Therefore, we should have $\lambda _{2}>|2\mathbf {z}_{j}^{T}\left (\mathbf {x}_{i}-\mathbf {X}_{-i}\boldsymbol {\beta }^{*}\right)+\lambda _{1}s_{1j}|$ and $\lambda _{2}>|2\tilde {\mathbf {z}}_{j}^{T}\left (\tilde {\mathbf {x}}_{i}-\tilde {\mathbf {X}}_{-i}\boldsymbol {\beta }^{*}\right)+\lambda _{1}\tilde {s}_{1j}|$, which can be satisfied if we choose $\lambda _{2}=\max _{j}\max \{\lambda _{1}+ |2\mathbf {z}_{j}^{T}\left (\mathbf {x}_{i}-\mathbf {X}_{-i}\boldsymbol {\beta }^{*}\right)|, \lambda _{1}+|2\tilde {\mathbf {z}}_{j}^{T}\left (\tilde {\mathbf {x}}_{i}-\tilde {\mathbf {X}}_{-i}\boldsymbol {\beta }^{*}\right)| \}$. Therefore, the maximum value of *λ*_2_ can be written as

15 $$\begin{aligned} \lambda_{2\max}&=\max_{j\ne i}\max\{\lambda_{1}+|2\mathbf{x}_{j}^{T}\left(\mathbf{x}_{i}-\mathbf{X}_{-i}\boldsymbol{\beta}^{*}\right)|,\lambda_{1}\\ &\quad+ |2\tilde{\mathbf{x}}_{j}^{T}\left(\tilde{\mathbf{x}}_{i}-\tilde{\mathbf{X}}_{-i}\boldsymbol{\beta}^{*}\right)| \}. \end{aligned} $$

To find *λ*_2 max_ from (15), we need to know ***β***^∗^. This can be done by solving the Lasso problem that minimizes $J(\boldsymbol {\beta }) = \parallel \mathbf {x}_{i}-\mathbf {X}_{-i}\boldsymbol {\beta }\parallel ^{2} + \parallel \tilde {\mathbf {x}}_{i} - \tilde {\mathbf {X}}_{-i}\boldsymbol {\beta }\parallel ^{2} +2\lambda _{1}\parallel \boldsymbol {\beta }\parallel _{1}$ using an efficient algorithm such as glmnet [37].

#### Stability selection

As mentioned earlier, parameter *λ*_1_ encourages sparsity in the inferred gene network, while *λ*_2_ induces sparsity in the changes of the network under two conditions. Generally, larger values of *λ*_1_ and *λ*_2_ induce a higher level of sparsity. Therefore, appropriate values of *λ*_1_ and *λ*_2_ need to be determined, which can be done with cross validation [37]. However, the nonzero entries of matrices **B** and $\tilde {\mathbf {B}}$, estimated with a specific pair of values of *λ*_1_ and *λ*_2_ determined by cross validation, may not be stable in the sense that small perturbation in the data may result in considerably different **B** and $\tilde {\mathbf {B}}$. We can employ an alternative technique, named stability selection [38], to select stable variables, as described in the following.

We first determine the maximum value of *λ*_1_, namely *λ*_1 max_, using the method described earlier, then choose a set of *k*_1_ values for *λ*_1_, denoted as $\mathcal {S}_{1}=\left \{\lambda _{1\max }, \alpha _{1}\lambda _{1\max }, \alpha _{1}^{2}\lambda _{1\max }, \ldots,\ \alpha _{1}^{k_{1}-1}\lambda _{1\max } \right \} $, where 0<*α*_1_<1. For each value $\lambda _{1}\in \mathcal {S}_{1}$, we find the maximum value of *λ*_2_, namely *λ*_2 max_(*λ*_1_), and then choose a set of *k*_2_ values for *λ*_2_, denoted $\mathcal {S}_{2}(\lambda _{1})=\{\lambda _{2\max }(\lambda _{1}), \alpha _{2}\lambda _{2\max }(\lambda _{1}), \ldots, \alpha _{2}^{k_{2}-1}\lambda _{2\max }(\lambda _{1}) \}$, where 0<*α*_2_<1. This gives a set of *K*=*k*_1_*k*_2_ pairs of (*λ*_1_,*λ*_2_). After we create the parameter space, for each (*λ*_1_,*λ*_2_) pair in the space, we randomly divide the data $(\mathbf {X}, \tilde {\mathbf {X}})$ into two subsets of equal size, and infer the network with our proximal gradient algorithm using each subset of the data. We repeat this process for *N* times, which yields 2*N* estimated network matrices, $\hat {\mathbf {B}}$ and $\hat {\tilde {\mathbf {B}}}$. Typically, *N*=50 is chosen.

Let $m_{ij}^{(k)}, \tilde m_{ij}^{(k)}$, and $\Delta m_{ij}^{(k)}$ be the number of nonzero $\hat {b}_{ij}$’s and $\hat {\tilde b}_{ij}$’s, and $(\hat {b}_{ij}-\hat {\tilde b}_{ij})$’s, respectively, obtained with the *k*th pair of (*λ*_1_,*λ*_2_). Then, $r_{ij}=\sum \nolimits _{k=1}^{K} m_{ij}^{(k)}/(NK), \tilde r_{ij}=\sum \nolimits _{k=1}^{K} \tilde m_{ij}^{(k)}/(NK)$, and $\Delta r_{ij}=\sum \nolimits _{k=1}^{K} \Delta m_{ij}^{(k)}/(NK)$ give the frequency of an edge from gene *j* to gene *i* detected under two conditions, and the frequency of the changes for an edge from gene *j* to gene *i*, respectively. A larger $r_{ij}, \tilde r_{ij}$, or *Δr*_*ij*_ indicates a higher likelihood that an edge from gene *j* to gene *i* exists, or the edge from gene *j* to gene *i* has changed. Therefore, we will use $r_{ij}, \tilde r_{ij}$ and *Δr*_*ij*_ to rank the reliability of the detected edges and the changes of edges, respectively. Alternatively, we can declare an edge from gene *j* to gene *i* exists if *r*_*ij*_≥*c* or $\tilde r_{ij}\ge c$; and similarly the edge between gene *j* to gene *i* has changed if *Δr*_*ij*_≥*c*, where *c* is constant and can be any value in [0.6,0.9] [38].

The software package in Additional file 1 includes computer programs that implement Algorithm 1, as well as stability selection and cross validation. The default values for parameters *α*_1_,*α*_2_,*k*_1_, and *k*_2_ in stability selection are 0.7, 0.8, 10, and 10, respectively. In cross validation, a set $\mathcal {S}_{1}$ of *k*_1_ values of *λ*_1_ and a set $\mathcal {S}_{2}(\lambda _{1})$ of *k*_2_ values of *λ*_2_ for each *λ*_1_ were created similarly, and the default values of *α*_1_,*α*_2_,*k*_1_, and *k*_2_ are 0.6952, 0.3728, 20, and 8, respectively.

### Software glmnet and lqa

Two software packages, glmnet and lqa, were used in computer simulations. The software glmnet [37] for solving the Lasso problem is available at https://cran.r-project.org/web/packages/glmnet. The software lqa [28] used in [27] for inferring multiple gene networks is available at https://cran.r-project.org/web/packages/lqa/.

## Results

### Computer simulation with linear regression model

We generated data from one of *p* pairs of linear regression models in (3) instead of all *p* pairs of simultaneous equations in (2), or equivalently (3), as follows. Without loss of generality, let us consider the first equation in (3). The goal was to estimate ***β***_1_ and $\tilde {\boldsymbol {\beta }}_{1}$, and then identify pairs ($\beta _{i1}, \tilde {\beta }_{i1}$), where $\beta _{i1}\neq \tilde {\beta }_{i1}$. Entries of *n*×(*p*−1) matrices **X**_−1_ and $\tilde {\mathbf {X}}_{-1}$ were generated independently from the standardized Gaussian distribution. In the first simulation setup, we chose *n*=100 and *p*−1=200. Taking into account the sparsity in ***β***_1_, we let 10% of ***β***_1_’s entries be nonzero. Therefore, twenty randomly selected entries of ***β***_1_ were generated from a random variable uniformly distributed over the intervals [0.5,1.5] and [−1.5,−0.5], and remaining entries were set to zero. Similarly, twenty entries of $\tilde {\boldsymbol {\beta }}_{1}$ were chosen to be nonzero. Since the two regression models are similar, meaning that most entries of $\tilde {\boldsymbol {\beta }}_{1}$ are identical to those of $\boldsymbol {\beta }_{1}, \tilde {\boldsymbol {\beta }}_{1}$ was generated by randomly changing 10 entries of ***β***_1_ as follows: 4 randomly selected nonzero entries were set to zero, and 6 randomly selected zero entries were changed to a value uniformly distributed over the intervals [0.5,1.5] and [−1.5,−0.5]. Of note, since the number of nonzero entries in ***β***_1_ is greater than the number of zero entries, the number of entries changed from zero to nonzero (which is 6) is greater than the number of entries changed from nonzero to zero (which is 4). The noise vectors **e**_1_ and $\tilde {\mathbf {e}}_{1}$ were generated from a Gaussian distribution with mean zero and variance *σ*^2^ varying from 0.01 to 0.05, 0.1, and 0.5, and then **x**_1_ and $\tilde {\mathbf {x}}_{1}$ were calculated from (3).

Simulated data $\mathbf {x}_{1}, \tilde {\mathbf {x}}_{1}, \mathbf {X}_{-1}$ and $\tilde {\mathbf {X}}_{-1}$ were analyzed with our ProGAdNet, lqa [28] and glmnet [37]. Since lqa was employed by [27], the results of lqa represent the performance of the network inference approach in [27]. The glmnet algorithm implements the Lasso approach in [39]. Both ProGAdNet and lqa estimate ***β***_1_ and $\tilde {\boldsymbol {\beta }}_{1}$ jointly by solving the optimization problem (4), but glmnet estimates ***β***_1_ and $\tilde {\boldsymbol {\beta }}_{1}$ separately, by solving the following two problems separately, $\hat {\boldsymbol {\beta }_{1}}=\text {arg min}_{\boldsymbol {\beta }_{1}}\{ \parallel \mathbf {x}_{1}-\mathbf {X}_{-1}\boldsymbol {\beta }_{1}\parallel ^{2} +\lambda _{1}\parallel \boldsymbol {\beta }_{1}\parallel _{1}$, and $\hat {\tilde {\boldsymbol {\beta }}}_{1}=\text {arg min}_{\tilde {\boldsymbol {\beta }}_{1}}\parallel \tilde {\mathbf {x}}_{1}-\tilde {\mathbf {X}}_{-1}\tilde {\boldsymbol {\beta }}_{1}\parallel ^{2}+\lambda _{2}\parallel \tilde {\boldsymbol {\beta }}_{1}\parallel _{1}$. The lqa algorithm uses a local quadratic approximation of the nonsmooth penalty term [40] in the objective function, and therefore, it cannot shrink variables to zero exactly. To alleviate this problem, we set $\hat {\beta }_{i1}=0$ if $|\hat {\beta }_{i1}|<10^{-4}$, and similarly $\hat {\tilde {\beta }}_{i1}=0$ if $|\hat {\tilde {\beta }}_{i1}|<10^{-4}$, where $\hat {\beta }_{i1}$ and $\hat {\tilde {\beta }}_{i1}$ represent the estimates of *β*_*i*1_ and $\tilde {\beta }_{i1}$, respectively. Five fold cross validation was used to determine the optimal values of parameters *λ*_1_ and *λ*_2_ in the optimization problem. Specifically, for ProGAdNet and lqa, the prediction error (PE) was estimated at each pair of values of *λ*_1_ and *λ*_2_, and the smallest PE along with the corresponding values of *λ*_1_ and *λ*_2_,*λ*_1 min_ and *λ*_2 min_, were determined. Then, the optimal values of *λ*_1_ and *λ*_2_ were the values corresponding to the PE that was two standard error (SE) greater than the minimum PE, and were greater than *λ*_1 min_ and *λ*_2 min_, respectively. For glmnet, the optimal values of *λ*_1_ and *λ*_2_ were determined separately also with the two-SE rule.

**Fig. 1:**
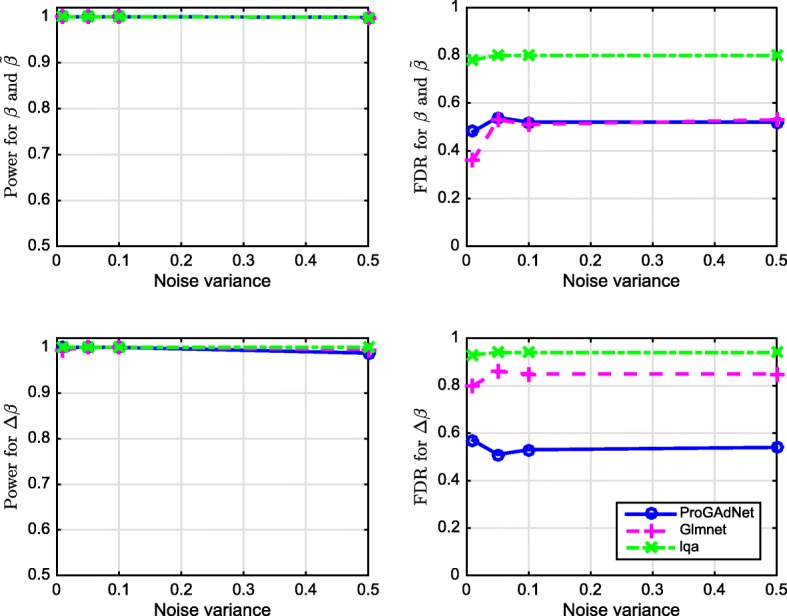
Performance of ProGAdNet, lqa, and Lasso in the inference of linear regression models. Number of samples *n*=100, and number of variables *p*−1=200

The inference process was repeated for 50 replicates of the data, and the detection power and the false discovery rate (FDR) for $(\boldsymbol {\beta }_{1},\tilde {\boldsymbol {\beta }}_{1})$ and $\Delta \boldsymbol {\beta }=\boldsymbol {\beta }_{1}-\tilde {\boldsymbol {\beta }}_{1}$ calculated from the results of the 50 replicates in the first simulation setup are plotted in Fig. 1. It is seen that all three algorithms offer almost identical power equal or close to 1, but exhibit different FDRs. The FDR of lqa is the highest, whereas the FDR of ProGAdNet is almost the same as that of glmnet for ***β***_1_ and $\tilde {\boldsymbol {\beta }}_{1}$, and the lowest for *Δ****β***_1_.

**Fig. 2:**
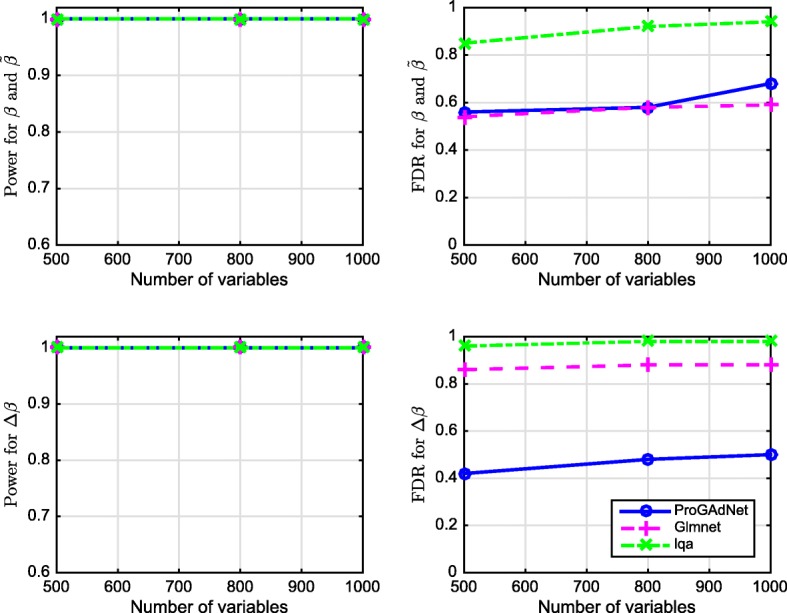
Performance of ProGAdNet, lqa, and Lasso in the inference of linear regression models. Number of samples *n*=150 and noise variance *σ*^2^=0.1

In the second simulation setup, we let sample size *n*=150, noise variance *σ*^2^=0.1, and the number of variables *p*−1 be 500, 800, and 1000. Detection power and FDR are depicted in Fig. 2. Again, the three algorithms have almost identical power, and ProGAdNet offers an FDR similar to that of glmnet, but lower than that of lqa for ***β***_1_ and $\tilde {\boldsymbol {\beta }}_{1}$, and the lowest FDR for *Δ****β***_1_. Simulation results in Figs. 1 and 2 demonstrate that our ProGAdNet offers the best performance when compared with glmnet and lqa. The CPU times of one run of ProGAdNet, lqa, and glmnet for inferring a linear model with *n*=150,*p*−1=1,000, and *σ*^2^=0.1 at the optimal values of *λ*_1_ and *λ*_2_ were 5.82, 145.15, and 0.0037 s, respectively.

### Computer Simulation with Gene Networks

The GeneNetWeaver software [41] was used to generate gene networks whose structures are similar to those of real gene networks. Note that GeneNetWeaver was also employed by the DREAM5 challenge for gene network inference to simulate golden standard networks [12]. GeneNetWeaver outputs an adjacency matrix to characterize a specific network structure. We chose the number of genes in the network to be *p*=50, and obtained a *p*×*p* adjacency matrix **A** through GeneNetWeaver. The number of nonzero entries of **A**, which determined the edges of the network, was 62. Hence the network is sparse, as the total number of possible edges is *p*(*p*−1)=2,450. We randomly changed 6 entries of **A** to yield another matrix $\tilde {\mathbf {A}}$ as the adjacency matrix of the gene network under another condition. Note that the number of changed edges is small relative to the number of existing edges.

After the two network topologies were generated, the next step was to generate gene expression data. Letting *a*_*ij*_ be the entry of **A** on the *i*th row and the *j*th column, we generated a *p*×*p* matrix **B** such that *b*_*ij*_=0 if *a*_*ij*_=0, and *b*_*ij*_ was randomly sampled from a uniform random variable on the intervals [−1,0) and (0,1] if *a*_*ij*_≠0. Another *p*×*p* matrix $\tilde {\mathbf {B}}$ was generated such that $\tilde {b}_{ij}=b_{ij}$ if $\tilde {a}_{ij}=a_{ij}$, or $\tilde {b}_{ij}$ was randomly generated from a uniform random variable on the intervals [−1,0) and (0,1] if $\tilde {a}_{ij}\ne a_{ij}$. Note that (2) gives **X**=**E**(**I**−**B**)^−1^ and $\tilde {\mathbf {X}}=\tilde {\mathbf {E}}(\mathbf {I}-\tilde {\mathbf {B}})^{-1}$. These relationships suggest generating first entries of **E** and $\tilde {\mathbf {E}}$ independently from a Gaussian distribution with zero mean and unit variance, and then finding matrices **X** and $\tilde {\mathbf {X}}$ from these two equations, respectively. With real data, gene expression levels **X** and $\tilde {\mathbf {X}}$ are measured with techniques such as microarray or RNA-seq, and there are always measurement errors. Therefore, we simulated measured gene expression data as **Y**=**X**+**V** and $\tilde {\mathbf {Y}}=\tilde {\mathbf {X}}+\tilde {\mathbf {V}}$, where **V** and $\tilde {\mathbf {V}}$ model measurement errors that were independently generated from a Gaussian distribution with zero mean and variance *σ*^2^ that will be specified later. Fifty pairs of network replicates and their gene expression data were generated independently.

Finally, gene networks were inferred with our ProGAdNet algorithm by solving the optimization problem (4), where $\mathbf {x}_{i}, \mathbf {X}_{-i}, \tilde {\mathbf {x}}_{i}$, and $\tilde {\mathbf {X}}_{-i}$ were replaced with the measured gene expression data $\mathbf {y}_{i}, \mathbf {Y}_{-i}, \tilde {\mathbf {y}}_{i}$, and $\tilde {\mathbf {Y}}_{-i}$. Stability selection was employed to rank the edges that were changed under two conditions. As comparison, we also used Lasso to infer the network topology under each condition by solving the following optimization problems

16 $$ \begin{aligned} \hat{\mathbf{B}}=&\text{arg min}_{\mathbf{B}}\parallel \mathbf{Y}-\mathbf{Y}\mathbf{B}\parallel^{2}+\lambda_{1} \parallel\mathbf{B}\parallel_{1} \\[-1pt] &\text {subject to~} b_{ii}=0, i=1,\ldots, p,\\[-1pt] \hat{\tilde{\mathbf{B}}}=&\text{arg min}_{\tilde{\mathbf{B}}}\parallel \tilde{\mathbf{Y}}-\tilde{\mathbf{Y}}\tilde{\mathbf{B}}\parallel^{2}+\lambda_{1} \parallel\tilde{\mathbf{B}}\parallel_{1} \\[-1pt] &\text {subject to~} \tilde{b}_{ii}=0, i=1,\ldots, p. \end{aligned}  $$

Note that each optimization problem can be decomposed into *p* separate problems that can be solved with Lasso. The glmnet algorithm [37] was again used to implement Lasso. The stability selection technique was employed again to rank the differential edges detected by Lasso. The lqa algorithm was not considered to infer simulated gene networks, because it is very slow and its performance is worse than ProGAdNet and Lasso as shown in the previous section. We also employed the GENIE3 algorithm in [42] to infer **B** and $\tilde {\mathbf {B}}$ separately, because GENIE3 gave the best overall performance in the DREAM5 challenge [12]. Finally, following the performance assessment procedure in [12], we used the precision-recall (PR) curve and the area under the PR curve (AUPR) to compare the performance of ProGAdNet with that of Lasso and GENIE3. For ProGAdNet and Lasso, the estimate of $\Delta \mathbf {B}=\mathbf {B}-\tilde {\mathbf {B}}$ was obtained, and the nonzero entries of *Δ***B** were ranked based on their frequencies obtained in stability selection. Then, the PR curve for changed edges was obtained from the ranked entries of *Δ***B** from pooled results for the 50 network replicates. Two lists of ranked network edges were obtained from GENIE3: one for **B** and the other for $\tilde {\mathbf {B}}$. For each cutoff value of the rank, we obtain an adjacency matrix **A** from **B** as follows: the (*i,j*)th entry of **A***a*_*ij*_=1 if *b*_*ij*_ is above the cutoff value, and otherwise *a*_*ij*_=0. Similarly, another adjacency matrix $\tilde {\mathbf {A}}$ was obtained from $\tilde {\mathbf {B}}$. Then, the PR curve for changed edges detected by GENIE3 was obtained from $\mathbf {A}-\tilde {\mathbf {A}}$, again from pooled results for the 50 network replicates.

**Fig. 3:**
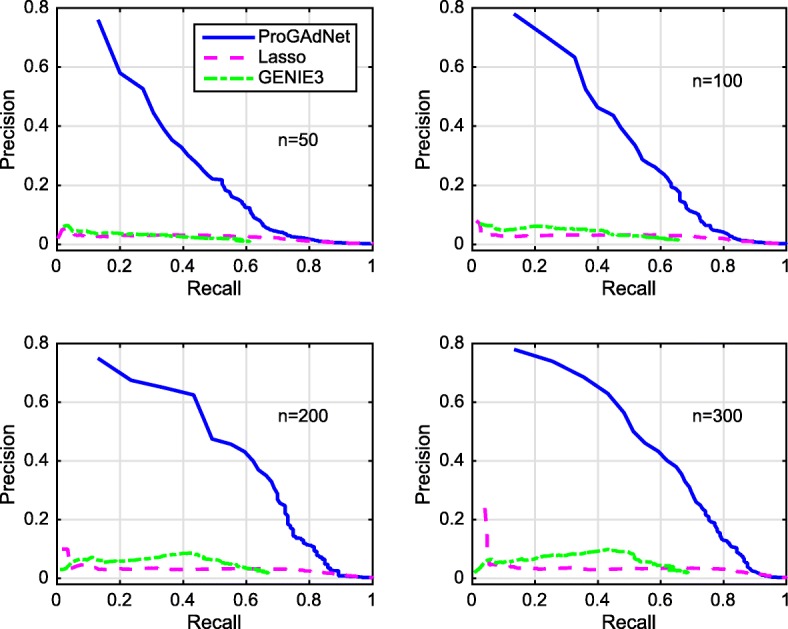
Precision-recall curves for ProGAdNet, Lasso, and GENIE3 in detecting changed edges of simulated gene networks. Variance of the measurement noise is *σ*^2^=0.05, and sample size *n*=50, 100, 200, and 300

**Fig. 4:**
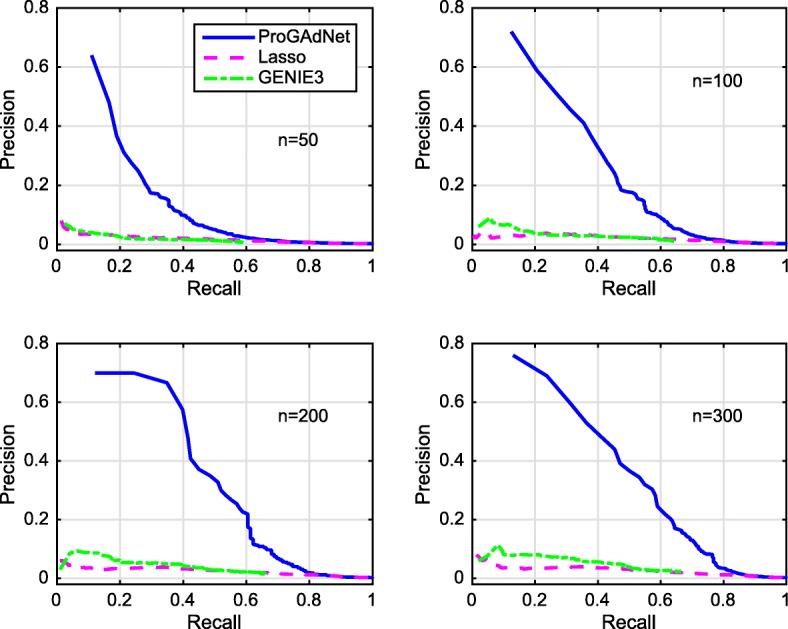
Precision-recall curves for ProGAdNet, Lasso, and GENIE3 in detecting changed edges of simulated gene networks. Variance of the measurement noise is *σ*^2^=0.5, and sample size *n*=50, 100, 200, and 300

Figures 3 and 4 depict the PR curves of ProGAdNet, Lasso, and GENIE3 for measurement noise variance *σ*^2^=0.05 and 0.5, respectively. The number of samples varies from 50, 100, 200 to 300. It is seen from Fig. 3 that our ProGAdNet offers much better performance than Lasso and GENIE3. When the noise variance increases from 0.05 to 0.5, the performance of all three algorithms degrades, but our ProGAdNet still outperforms Lasso and GENIE3 considerably, as shown in Fig. 4. Table 1 lists AUPRs of ProGAdNet, Lasso and GENIE3, which again shows that our ProGAdNet outperforms Lasso and GENIE3 consistently at all sample sizes.

**Table 1:** AUPRs of ProGAdNet, Lasso, and GENIE3 for detecting the changed edges of simulated gene networks

	*σ*^2^=0.05			*σ*^2^=0.5		
# samples	ProGAdNet	Lasso	GENIE3	ProGAdNet	Lasso	GENIE3
50	0.206	0.023	0.018	0.106	0.018	0.014
100	0.288	0.025	0.028	0.202	0.021	0.022
200	0.356	0.030	0.039	0.280	0.024	0.031
300	0.380	0.031	0.044	0.289	0.026	0.038

### Analysis of breast cancer data

We next use the ProGAdNeT algorithm to analyze RNA-seq data of breast tumors and normal tissues. In The Cancer Genome Atlas (TCGA) database, there are RNA-seq data for 1098 breast invasive carcinoma (BRCA) samples and 113 normal tissues. The RNA-seq level 3 data for 113 normal tissues and their matched BRCA tumors were downloaded. The TCGA IDs of these 226 samples are given in Additional file 2. The scaled estimates of gene expression levels in the dataset were extracted, and they were multiplied by 10^6^, which yielded transcripts per million value of each gene. The batch effect was corrected with the removeBatchEffect function in the Limma package [43] based on the batch information in the TCGA barcode of each sample (the “plate” field in the barcode). The RNA-seq data include expression levels of 20,531 genes. Two filters were used to obtain informative genes for further network analysis. First, genes with their expression levels in the lower 30 percentile were removed. Second, the coefficient of variation (CoV) was calculated for each of the remaining genes, and then genes with their CoVs in the lower 70 percentile were discarded. This resulted in 4310 genes, and their expression levels in 113 normal tissues and 113 matched tumor tissues were used by the ProGAdNet algorithm to jointly infer the gene networks in normal tissues and tumors, and then to identify the difference in the two gene networks. The list of the 4310 genes is in Additional file 3, and their expression levels in tumors and normal tissues are in two data files in the software package in Additional file 1.

Since small changes in *b*_*ji*_ in the network model (1) may not have much biological effect, we regarded the regulatory effect from gene *j* to gene *i* to be changed using the following two criteria rather than the simple criterion $\tilde b_{ji}\ne b_{ji}$. The first criterion is $|\tilde b_{ji}- b_{ji} |\ge \min \{ |\tilde b_{ji}|, |b_{ji}|\}$, which ensures that there is at least one-fold change relative to $\min \{ |\tilde b_{ji}|, |b_{ji}|\}$. However, when one of $\tilde b_{ji}$ and *b*_*ji*_ is zero or near zero, this criterion does not filter out very small $|\tilde b_{ji}- b_{ji} |$. To avoid this problem, we further considered the second criterion. Specifically, nonzero $\tilde b_{ji}$ and *b*_*ji*_ for all *j* and *i* were obtained, and the 20 percentile value of all $|\tilde b_{ji}|$ and |*b*_*ji*_|, *T*, was found. Then, the second criterion is $\max \{|\tilde b_{ji}|, |b_{ji}|\}\ge T$. As in computer simulations, the stability selection was employed to identify network changes reliably. As the number of genes, 4310, is quite big, it is time consuming to repeat 100 runs per *λ*_1_ and *λ*_2_ pair. To reduce the computational burden, we used five-fold cross validation to choose the optimal values of *λ*_1_ and *λ*_2_ based on the two-SE rule used in computer simulation, and then performed stability selection with 100 runs for the pair of optimal values. Note that stability selection at an appropriate point of hyperparameters is equally valid compared with that done along a path of hyperparameters [38]. The threshold for *Δr*_*ij*_ for determining network changes as described in the Method section was chosen to be *c*=0.9.

Our network analysis with ProGAdNeT identified 268 genes that are involved in at least one changed edge. Names of these genes are listed in Additional file 4. We named the set of these 268 genes as the dNet set. We also extracted the raw read count of each gene from the RNA-seq dataset and employed DESeq2 [44] to detect the differentially expressed genes. The list of 4921 differentially expressed genes detected at FDR < 0.001 and fold change ≥1 is also in Additional file 4. Among 268 dNet genes, 196 genes are differentially expressed, and the remaining 72 genes are not differentially expressed, as shown in Additional file 4.

To assess whether the dNet genes relate to the disease status, we performed gene set enrichment analysis (GSEA) with the C2 gene sets in the molecular signatures database (MSigDB) [45, 46]. C2 gene sets consist of 3777 human gene sets that include pathways in major pathway dabases such as KEGG [47], REACTOME [48], and BIOCARTA [49]. After excluding gene sets with more than 268 genes or less than 15 genes, 2844 gene sets remained. Of note, the default value for the minimum gene set size at the GSEA website is 15. Here we also excluded the gene sets whose size is greater than 268 (the size of the dNet set), because large gene sets may tend to be enriched in a small gene set by chance. Searching over the names of these 2844 gene sets with key words “breast cancer”, “breast tumor”, “breast carcinoma” and “BRCA” through the “Search Gene Sets” tool at the GSEA website identified 258 gene sets that are related to breast cancer. Using Fisher’s exact test, we found that 121 of 2844 C2 gene sets were enriched in the dNet gene set at a FDR of < 10^−3^. The list of the 121 gene sets is in Additional file 5. Of these 121 gene sets, 31 are among the 258 breast cancer gene sets, which is highly significant (Fisher’s exact test *p*-value 2×10^−7^). The top 20 enriched gene sets are listed in Table 2. As seen from names of these gene sets, 11 of the 20 gene sets are breast cancer gene sets, and 7 sets are related to other types of cancer. These GSEA results clearly show that the dNet gene set that our ProGAdNet algorithm identified is very relevant to the breast cancer.

**Table 2:** Top 20 MSigDB C2 gene sets that are enriched in the dNet gene set identified from the BRCA data

	

Eleven gene sets related to BRCA are highlighted

### Analysis of kidney cancer data

We also analyzed another dataset in the TCGA database, the kidney renal clear cell carcinoma (KIRC) dataset, which contains the RNA-seq data of 463 tumors and 72 normal tissues. The RNA-seq level 3 data for the 72 normal tissues and their matched tumors were downloaded. The TCGA IDs of these 144 samples are given in Additional file 6. We processed the KIRC data in the same way as in processing the BRCA data. After the two filtering steps, we again got expression levels of 4310 genes. The list of the 4310 genes is in Additional file 7, and their expression levels in 72 tumors and 72 normal tissues are in two data files in Additional file 1.

Analysis of the KIRC data with ProGAdNet identified 1091 genes that are involved in at least one changed edge. We chose the top 460 genes that are involved in at least 3 changed edge to do further GSEA. Names of these 460 genes are listed in Additional file 8. We named the set of these 460 genes as the dNetK set. We also extracted the raw read count of each gene from the RNA-seq dataset and employed DESeq2 [44] to detect the differentially expressed genes. The list of 5432 differentially expressed genes detected at FDR < 0.001 and fold change ≥1 is also in Additional file 8. Among 460 dNetK genes, 395 genes are differentially expressed, and the remaining 65 genes are not differentially expressed, as shown in Additional file 8.

After excluding genes sets with more than 460 genes or less than 15 genes from the 3777 human C2 gene sets in MSigDB, we obtained 3019 gene sets for GSEA. Using Fisher’s exact test, we found 251 of the 3019 C2 gene sets were enriched in the dNetK set of 460 genes at a FDR of < 10^−3^. The list of the 251 gene sets is in Additional file 9. The top 20 enriched gene sets are listed in Table 3. Among the top 20 gene sets, 2 gene sets are related to kidney diseases, 8 gene sets are related to several different types of cancer, and 5 gene sets are related to the immune system. The 460 genes were ranked according to the number of changed edges that these genes are involved in, and the top 10 genes are NADH Dehydrogenase (Ubiquinone) 1 Alpha Subcomplex 4-Like 2 (NDUFA4L2), Uromodulin (UMOD), Angiopoietin-Like 4 (ANGPTL4), Nicotinamide N-methyltransferase (NNMT), Carbonic anhydrase 9 (CA9), Insulin-like growth factor binding protein 3 (IGFBP3), Apolipoprotein E/C1 (APOE/C1), complement component 3 (C3), vimentin (VIM), and complement C4A. Eight of the top 10 genes except C3 and C4A have been reported to be implicated in renal cell carcinoma (RCC), as discussed in the next section.

**Table 3:** Top 20 MSigDB C2 gene sets that are enriched in the dNetK gene set identified from the KIRC data

	

Eight gene sets related to cancer are highlighted

## Discussion

Computer simulations demonstrated that our ProGAdNet significantly outperformed three other algorithms, glmnet, GENIE3, and lqa, in detecting network changes. The performance gain of ProGAdNet over glmnet and GENIE3 is expected, because glmnet and GENIE3 infer two gene networks separately, while ProGAdNet infers two networks jointly and takes into account the similarity between two networks. Although ProGAdNet and lqa solve the same optimization problem, ProGAdNet significantly outperforms lqa. The performance gap is due to the fact that the lqa algorithm uses an approximation of the objective function, whereas our algorithm solves optimization problem (4) exactly. In other words, our ProGAdNet algorithm can always find the optimal solution to the optimization problem, since the objective function is convex, but the lqa algorithm generally cannot find the optimal solution. Moreover, our computer simulations show that our ProGAdNet algorithm is much faster than the lqa algorithm.

As mentioned earlier, eight of the top 10 genes in the differential gene network of KIRC except C3 and C4A have been reported to be implicated in renal cell carcinoma (RCC). Specifically, NDUFA4L2 is overexpressed in clear cell RCC (ccRCC); its mRNA level is correlated with tumor stage and overall survival time [50, 51]; and the association of NDUFA4L2 with ccRCC is regulated by ELK1 [52]. UMOD expression is downregulated in RCC [53, 54], and the homozygous genotype of UMOD is associated with more aggressive RCC [55]. ANGPTL4 plays an important role in several cancers [56–60]. It has been showed that the serum ANGPTL4 level in RCC patients were higher than the level in patients with other types of solid tumor, such as bladder cancer, breast cancer, gastrointestinal cancer and lung adenocarcinoma, suggesting that the serum ANGPTL4 may be a diagnostic and prognostic biomarker for RCC [61]. NNMT is over-expressed in RCC; its high expression level is significantly associated with unfavorable prognosis of RCC [62]; and it can potentially serve as a biomarker for early detection of RCC [63]. CA9 is a transmembrane member of the carbonic anhydrase family. It is not expressed in healthy renal tissue, but is overexpressed in most ccRCC; it is a promising molecular marker for diagnosis, prognosis and therapy of CCRCC [64, 65]. IGFBP3 shows overexpression in association with markers of poor prognosis in many tumour types [66], including RCC [67]. Single nucleotide polymorphisms (SNPs) at the APOE/C1 locus are found to be associated with RCC risk [68]. The expression of vimentin was upregulated significantly in RCC [69, 70], and the expression level of vimetin was positively correlated with the pathological grade and clinical stage of RCC [70]. These results show that the dNetK gene set that our ProGAdNet algorithm identified from the KIRC dataset is informative about the cancer disease status.

## Conclusion

In this paper, we developed a very efficient algorithm, named ProGAdNet, for inference of two gene networks based on gene expression data under two different conditions, which were further used to identify differential changes in the network. Computer simulations showed that our ProGAdNet offered much better inference accuracy than existing algorithms. Analysis of a set of RNA-seq data of breast tumors and normal tissues with ProGAdNet identified a set of genes involved in differential changes of the gene network. A number of gene sets of breast cancer or other types of cancer are significantly enriched in the identified gene set. Network analysis of a kidney cancer dataset also identified a set of genes involved in network changes, and the majority of the top genes identified have been reported to be implicated in kidney cancer. These results show that the identified gene sets are very informative about the disease status of the tissues. As gene network rewiring occurs frequently under different molecular context, our ProGAdNet algorithm provides a valuable tool for identifying changed gene-gene interactions.

## Additional files

Additional file 1 Software package for the ProGAdNet algorithm and computer simulations. (ZIP 8786 kb)

Additional file 2 The list of the TCGA IDs of 113 BRCA and 113 normal tissue samples. (XLSX 10 kb)

Additional file 3 The list of 4310 genes whose expression levels in BRCA and normal tissues are analyzed by ProGAdNet. The two files for the expression levels of these genes in tumors and normal tissues are in the software package in Additional file 1. (XLSX 78 kb)

Additional file 4 The list of 268 genes that are involved in at least one changed network edges identified from the gene expression data of BRCA and normal tissues. Also included is the list of differentially expressed genes in BRCA. (XLSX 582 kb)

Additional file 5 The list of 121 MSigDB C2 gene sets that are significantly enriched in the set of 268 dNet genes in Additional file 4 identified from the BRCA data. (XLSX 15 kb)

Additional file 6 The list of the TCGA IDs of 72 KIRC and 72 normal tissue samples. (XLSX 6.55 kb)

Additional file 7 The list of 4310 genes whose expression levels in KIRC and normal tissues are analyzed by ProGAdNet. The two files for the expression levels of these genes in tumors and normal tissues are in the software package in Additional file 1. (XLSX 76 kb)

Additional file 8 The list of 460 genes that are involved in at least three changed network edges identified from the gene expression data of KIRC and normal tissues. Also included is the list of differentially expressed genes in KIRC. (XLSX 646 kb)

Additional file 9 The list of 251 MSigDB C2 gene sets that are significantly enriched in the set of 460 dNetK genes in Additional file 8 identified from the KIRC data. (XLSX 22 kb)

## Abbreviations

AUPR: Area under the PR curve
BRCA: Breast invasive carcinoma
CoV: Coefficient of variation
DiNA: Differential network analysis
FDR: False discovery rate
FLSA: Fused Lasso signal approximator
GGM: Gaussian graphical model
GSEA: Gene set enrichment analysis
KIRC: Kidney renal clear cell carcinoma
PE: Prediction error
PR: Precision-recall
ProGAdNet: proximal gradient algorithm for differential network
RCC: Renal cell carcinoma
SE: Standard error
SNP: Single nucleotide polymorphism TCGA: The cancer genome atlas
TF: Transcription factor
